# Assessing physical activity in people with mental illness: 23-country reliability and validity of the simple physical activity questionnaire (SIMPAQ)

**DOI:** 10.1186/s12888-020-2473-0

**Published:** 2020-03-06

**Authors:** S. Rosenbaum, R. Morell, A. Abdel-Baki, M. Ahmadpanah, T. V. Anilkumar, L. Baie, A. Bauman, S. Bender, J. Boyan Han, S. Brand, S. Bratland-Sanda, J. Bueno-Antequera, A. Camaz Deslandes, L. Carneiro, A. Carraro, C. P. Castañeda, F. Castro Monteiro, J. Chapman, J. Y. Chau, L. J. Chen, B. Chvatalova, L. Chwastiak, G. Corretti, M. Dillon, C. Douglas, S. T. Egger, F. Gaughran, M. Gerber, E. Gobbi, K. Gould, M. Hatzinger, E. Holsboer-Trachsler, Z. Hoodbhoy, C. Imboden, P. S. Indu, R. Iqbal, F. R. Jesus-Moraleida, S. Kondo, P. W. Ku, O. Lederman, E. H. M. Lee, B. Malchow, E. Matthews, P. Mazur, A. Meneghelli, A. Mian, B. Morseth, D. Munguia-Izquierdo, L. Nyboe, B. O’Donoghue, A. Perram, J. Richards, A. J. Romain, M. Romaniuk, D. Sadeghi Bahmani, M. Sarno, F. Schuch, N. Schweinfurth, B. Stubbs, R. Uwakwe, T. Van Damme, E. Van Der Stouwe, D. Vancampfort, S. Vetter, A. Waterreus, P. B. Ward

**Affiliations:** 1 School of Psychiatry, UNSW Sydney, Sydney, Australia; 2 Centre de Recherche du Centre Hospitalier de l’Université de Montréal (CRCHUM), Montreal, Canada; 3 Behavioral Disorders and Substances Abuse Research Center, Hamadan University of Medical Sciences, Hamadan, Iran; 4 Department of Psychiatry, Government Medical College, Trivandrum, India; 5 Department of Psychosomatics and Psychotherapy, University Hospital Münster, Münster, Germany; 6 School of Public Health, University of Sydney, Sydney, Australia; 7 LWL-Klinik Marsberg, Hospital for Psychiatry, Psychotherapy and Psychosomatics, Marsberg, Germany; 8 California State University, Los Angeles, USA; 9 University of Basel, Psychiatric Clinics, Center for Affective, Stress and Sleep Disorders, Basel, Switzerland; 10 Department of Biomolecular Sciences, University of Urbino, Urbino, Italy; 11 The Sutherland Hospital, South Eastern Sydney Local Health District, Sydney, Australia; 12 Department of Sport, Physical Education and Outdoor Studies, University of South-Eastern Norway, Bø, Notodden, Norway; 13 Physical Performance & Sports Research Center, Department of Sports and Computer Science, Section of Physical Education and Sports, Faculty of Sports Sciences, Universidad Pablo de Olavide, Seville, Spain; 14 Psychiatry Institute, Universidade Federal do Rio de Janeiro, Rio de Janeiro, Brazil; 15 Research Centre in Sports Sciences, Health Sciences and Human Development, CIDESD, GERON Research Community, Vila Real, Portugal; 16 Faculty of Education, Free University of Bolzano, Bolzano, Italy; 17 Early Intervention Program, JHorwitz Psychiatric Institute, Santiago, Chile; 18 Hospital de Clínicas de Porto Alegre, Universidade Federal do Rio Grande do Sul, Porto Alegre, Brazil; 19 QIMR Berghofer Medical Research Institute, Brisbane, Australia; 20 Department of Health Systems and Populations, Macquarie University, Sydney, Australia; 21 Department of Exercise Health Science, National Taiwan University of Sport, Taichung, Taiwan; 22 National Institute of Mental Health, Klecany, Czech Republic; 23 Department of Psychiatry and Behavioral Sciences, University of Washington, Seattle, USA; 24 Department of Mental Health, North-West Tuscany, Italy; 25 HSE Louth Meath Mental Health Services, Louth, Ireland; 26 South Coast Private Hospital, Wollongong, Australia; 27 Department of Psychiatry, Faculty of Medicine, University of Oviedo, Oviedo, Spain; 28 Department of Psychiatry, Psychotherapy and Psychosomatics, University Hospital of Psychiatry Zurich, University of Zurich, Zurich, Switzerland; 29 South London and Maudesley NHS Foundation Trust, London, UK; 30 Department of Sport, Exercise and Health, Division of Sport and Psychosocial Health, University of Basel, Basel, Switzerland; 31 St John of God Hospital, North Richmond, Australia; 32 Psychiatric Services Solothurn, Solothurn, Switzerland; 33 Adult Psychiatric Clinics (UPKE), University of Basel, Basel, Switzerland; 34 Department of Paediatrics and Child Health, The Aga Khan University, Karachi, Pakistan; 35 Private Clinic Wyss, Muenchenbuchsee, Switzerland; 36 Department of Community Medicine, Government Medical College, Trivandrum, India; 37 Department of Community Health Sciences, Aga Khan University, Karachi, Pakistan; 38 Department of Physical Therapy, Universidade Federal do Ceará, Fortaleza, Brazil; 39 Department of Neuropsychiatry, The University of Tokyo Hospital, Tokyo, Japan; 40 Graduate Institute of Sports and Health, National Changhua University of Education, Changhua, Taiwan; 41 Keeping the Body In Mind, South Eastern Sydney Local Health District, Sydney, Australia; 42 Department of Psychiatry, University of Hong Kong, Hong Kong, China; 43 Department of Psychiatry and Psychotherapy, University Medical Center Göttingen, Göttingen, Germany; 44 School of Health Sciences, Waterford Institute of Technology, Waterford, Ireland; 45 Association of early intervention in mental disorders-Cambiare la Rotta-Onlus, Milano, Italy; 46 Department of Psychiatry, Aga Khan University, Karachi, Pakistan; 47 School of Sport Sciences, UiT The Arctic University of Norway, Tromsø, Norway; 48 Department of Affective Disorders, Aarhus University Hospital, Aarhus, Denmark; 49 Orygen, the National Centre of Excellence in Youth Mental Health, Melbourne, Australia; 50 Faculty of Health, Victoria University Wellington, Wellington, New Zealand; 51 Gallipoli Medical Research Institute, Brisbane, Australia; 52 Kermanshah University of Medical Sciences, Sleep Disorders and Substance Abuse Prevention Research Center, Kermanshah, Iran; 53 Department of Sports Methods and Techniques, Federal University of Santa Maria, Santa Maria, Brazil; 54 Department of Psychological Medicine, King’s College London, London, England; 55 Faculty of Medicine, Nnamdi Azikiwe University, Awka, Nigeria; 56 Department of Rehabilitation Sciences, KU Leuven, Leuven, Belgium; 57 University of Groningen, University Medical Center Groningen, University Center of Psychiatry, Groningen, Netherlands; 58 Neuropsychiatric Epidemiology Research Unit, School of Population and Global Health, University of Western Australia, Perth, Australia; 59 Schizophrenia Research Unit, Ingham Institute of Applied Medical Research, Liverpool, Australia

**Keywords:** Physical activity, Measurement, Mental illness, Exercise, Assessment, Sedentary behaviour

## Abstract

**Background:**

Physical inactivity is a key contributor to the global burden of disease and disproportionately impacts the wellbeing of people experiencing mental illness. Increases in physical activity are associated with improvements in symptoms of mental illness and reduction in cardiometabolic risk. Reliable and valid clinical tools that assess physical activity would improve evaluation of intervention studies that aim to increase physical activity and reduce sedentary behaviour in people living with mental illness.

**Methods:**

The five-item Simple Physical Activity Questionnaire (SIMPAQ) was developed by a multidisciplinary, international working group as a clinical tool to assess physical activity and sedentary behaviour in people living with mental illness. Patients with a DSM or ICD mental illness diagnoses were recruited and completed the SIMPAQ on two occasions, one week apart. Participants wore an Actigraph accelerometer and completed brief cognitive and clinical assessments.

**Results:**

Evidence of SIMPAQ validity was assessed against accelerometer-derived measures of physical activity. Data were obtained from 1010 participants. The SIMPAQ had good test-retest reliability. Correlations for moderate-vigorous physical activity was comparable to studies conducted in general population samples. Evidence of validity for the sedentary behaviour item was poor. An alternative method to calculate sedentary behaviour had stronger evidence of validity. This alternative method is recommended for use in future studies employing the SIMPAQ.

**Conclusions:**

The SIMPAQ is a brief measure of physical activity and sedentary behaviour that can be reliably and validly administered by health professionals.

## Background

People with mental disorders experience high rates of comorbid chronic physical diseases including diabetes, obesity, and cardiovascular disease, contributing to an increased mortality risk, regardless of psychiatric diagnosis [1, 2]. Although genetic factors contribute to overall cardio-metabolic risk, the role of modifiable lifestyle behaviours, such as physical inactivity and low physical fitness are becoming better recognised [3, 4]. Increasing physical activity remains a cornerstone of metabolic and cardiovascular disease treatment and prevention in the general population [5], with growing recognition that cardiorespiratory fitness is inversely associated with all-cause mortality [6]. A 2019 Lancet Psychiatry Commission on protecting the physical health of people with mental illness recommended that physical activity be incorporated as part of routine psychiatric care regardless of diagnosis and across all treatment settings [7]. In addition to the established physical health benefits, physical activity can have both preventive and treatment effects on psychiatric symptomatology for people experiencing a range of mental disorders, including depression [8–10], anxiety disorders [11] and psychosis [12].

People with mental disorders have been shown to be significantly less physically active or less likely to meet international physical activity recommendations [4, 13–15]. Despite numerous calls for physical activity to be recognised as an integral component of routine psychiatric care [16], including recognition in the recent WHO guidelines [17], access to programs and integration within mental health services remains ad-hoc in many jurisdictions, with limited funding or resources available for implementation in routine clinical care [18].

One barrier to the implementation of physical activity programs within mental health settings is the lack of a clinical tool to assess physical activity that enables risk stratification based on activity levels. Similarly, without a clinically feasible tool that can be used as part of routine care, evaluating the effectiveness of interventions designed to increase physical activity is problematic. Currently methods used to assess physical activity vary in cost, accuracy and feasibility [19].

Furthermore, no self-reported physical activity measures have been developed specifically for people with mental illness and there is little consensus regarding the utility of existing self-report questionnaires. A 2014 review of the psychometric properties of physical activity assessment tools identified 10 unique self-report questionnaires that had been used in psychiatric populations with limited evidence for robust psychometric properties [20]. Arguably, the most commonly used questionnaire for research purposes is the International Physical Activity Questionnaire (IPAQ). The IPAQ was developed in 2003 specifically for assessing population levels of total physical activity and allowing for cross-country comparison [21, 22]. In 2006, the measurement properties of the IPAQ (short-form) in 35 people with schizophrenia who were living in the community, were found to be comparable to those in the general population [23]. The IPAQ has been used extensively to measure physical activity in people diagnosed with mental health conditions [24] including as a measure of change in clinical intervention studies. The validity of the IPAQ to assess total sedentary behaviour has also been questioned, with recent data suggesting that the IPAQ is unsuitable for population level assessment of sitting time among individuals with schizophrenia [25]. Furthermore, a recent study using data from the UK Biobank found that, although people with schizophrenia self-reported the same physical activity levels as the general population assessed using the IPAQ, objective measures revealed that they were overall less active than 80% of the general population, providing evidence that existing self-report measures used in epidemiological studies of physical activity may fail to capture lower physical activity levels in schizophrenia [26].

Use of the IPAQ in clinical settings may also be problematic for a number of reasons and differs from the intended purpose of the tool which was to conduct population surveillance [22]. For example, physical activity lasting less than 10 min is not assessed using self-report questionnaires such as the IPAQ, despite the potential mental health benefits of such activity. The Second Edition of the Physical Activity Guidelines for Americans published in 2018, note that any amount of physical activity has some health benefits, and removed the recommendation that only 10-min bouts of physical activity counted towards meeting the guidelines [27]. Finally, while the IPAQ assesses total levels of physical activity, it does not differentiate between activities performed for the purposes of structured exercise and physical activities performed as part of daily life, which may also have important implications for mental health outcomes [28].

The measurement of physical activity in people with mental illness presents unique challenges given diagnostic heterogeneity and differing symptom profiles among psychiatric patients. For example, clinical variability in mood may influence the ability to accurately respond to self-report questionnaires, especially among people who experience symptom fluctuations such as those with rapid-cycling bipolar disorder. Psychotic symptoms, grandiosity, and severe symptoms of depression and anxiety are also likely to influence the utility of self-report measures. In addition, people with mental illness may have unique barriers to accessing exercise facilities such that hospitalization may result in restricted opportunities to engage in physical activity. Alternatively, inpatient admission may allow access to customised physical activity interventions in some settings. Given that physical activity is a key strategy to prevent cardio-metabolic disease [17], a leading cause of premature mortality in people with mental illness, a measure appropriate for routine clinical use in this population is required.

In order to ensure the accurate assessment of physical activity across people with mental illness, we developed a self-report, physical activity measurement tool, designed to be administered via interview. The Simple Physical Activity Questionnaire (SIMPAQ) is a tool suitable for routine clinical use, and the current study was conducted to determine the reliability and validity of the SIMPAQ for assessing physical activity among inpatients and outpatients experiencing mental illness.

## Method

Approval was obtained from the Human Research Ethics Committee (HREC) of UNSW Sydney, Australia (HC15586) as the lead site. In addition, local ethics approval was sought from each participating site as per local requirements. Details of approving committees are provided under the Declaration section below.

### SIMPAQ development

The SIMPAQ was iteratively developed between April 2014 and May 2016 by a multidisciplinary, international working group with both clinical and research expertise (including psychiatrists, psychologists, physiotherapists, exercise physiologists, and epidemiologists) regarding physical health care interventions for people living with mental illness. The first meeting was held in Padua, Italy, in April, 2014, to identify the common challenges experienced when assessing physical activity among people with mental illness. At a subsequent meeting in July, 2015, held at the Institute of Psychiatry, Psychology and Neuroscience in London (UK), consensus agreement on the wording of the questions that constitute the SIMPAQ was obtained.

### Participating Research sites

In addition to disseminating information about the project via the international workgroup, an editorial was published in 2016 describing the proposed validation process that helped to identify additional study sites [29]. All study material and administration protocols were available from the project website (www.simpaq.org) when recruitment commenced in May 2016. All sites were required to nominate a site coordinator and sign an authorship agreement document. Along with study material, site coordinators received a briefing from investigators SR and PBW and were also in regular contact with the study coordinator RM. Eligibility criteria for potential sites included willingness to recruit patients meeting the inclusion criteria outlined below and availability of a site coordinator with expertise in either mental health or physical activity research.

### Translation process

Translation was conducted according to the Principles of Good Practice for the Translation and Cultural Adaptation Process for Patient-Reported Outcomes (PRO) Measures, as proposed by the International Society of Pharmacoeconomics and Outcomes (ISPOR) [30]. This process involved ten steps including 1) preparation, 2) forward translation, 3) reconciliation, 4) back translation, 5) back translation review, 6) harmonization, 7) cognitive debriefing, 8) review of cognitive debriefing results and finalization, 9) proof reading, 10) publication on SIMPAQ website.

### Participants

All participants were required to provide written informed consent and be willing to wear an accelerometer for seven days. Eligibility criteria also included: i) aged between 18 and 65 years, ii) a current inpatient or outpatient of one of the treatment facilities identified as a SIMPAQ validation study site and iii) met DSM-5 or ICD-10 criteria for any mental disorder, excluding eating disorders.

### Study procedures

Participants were approached by a researcher nominated by the site coordinator who was not involved in the direct care of the patient. The researcher obtained written informed consent. Data was collected from each participant during two face-to-face sessions, at least seven days apart. Researchers involved in data collection included either mental health or exercise professionals.

#### Session 1

Demographic and descriptive information was collected including assessment of symptoms and cognitive ability. Participants completed the SIMPAQ (Time 1) and were given a tri-axial accelerometer (Actigraph GT3x or GT3x + (both models contain the same accelerometer and processing method)) along with standardised instructions for wearing the device.

#### Session 2

Participants completed the SIMPAQ (Time 2) covering the period of accelerometer wear time.

### Data collection

#### Participant demographics and descriptive information

A standardised form was used to obtain demographic and descriptive information including: age, sex, treatment setting (inpatient or other), years of completed education, previous 7-day employment status (yes or no), previous 7-day tobacco smoking status (yes or no), body mass index (derived from measures of height [m] and weight [kg]).

Each country in which a site acquired SIMPAQ data was assigned an income status (either *high income* or *other)* based on World Bank classification (www.worldbank.org).

#### Psychiatric diagnoses

Psychiatric diagnoses that applied to individual participants based on medical records were recorded. It was recognized that participants may meet criteria for more than one psychiatric diagnosis, and all diagnoses that applied to each participant were recorded. The standardised form asked researchers to tick yes or no for the following diagnostic categories based on clinical diagnoses; schizophrenia spectrum disorders, bipolar disorder, depressive disorders, anxiety disorders, obsessive-compulsive disorders, substance-related & addictive disorders, neurocognitive disorders and other disorders. We identified individuals who were assigned a single diagnostic category, and those with psychiatric co-morbidity.

#### Physical health conditions

The presence or absence (yes or no) of the following physical health conditions at the time of assessment were also recorded by the researcher; diabetes, high cholesterol, high blood pressure, stroke and chronic pain based on self-report and medical records.

#### Medication status

Researchers were asked to indicate whether participants were currently prescribed the following classes of psychotropic medication (yes or no): antidepressant, antipsychotic, or mood stabilising medications.

#### Symptom severity – DSM-5 self rated level 1 cross cutting symptom measure

The 23-item DSM-5 Self-rated Level 1 Cross-cutting Symptom Measure [31] was used to assess symptom severity. This measure consists of 23 questions that assess 13 psychiatric domains, including depression, anger, mania, anxiety, somatic symptoms, suicidal ideation, psychosis, sleep problems, memory, repetitive thoughts and behaviours, dissociation, personality functioning, and substance use [31]. Each question asks about how much (or how often) the individual has been bothered by the specific symptom during the past two weeks and is rated on a 5-point scale (0 = none or not at all; 1 = slight or rare, less than a day or two; 2 = mild or several days; 3 = moderate or more than half the days; and 4 = severe or nearly every day). We summed the total scores across these domains and dichotomized the scores around the median (20); lower symptom severity was defined as scores < 21; higher symptom severity was defined as scores > = 21.

#### Cognitive functioning – Montreal cognitive assessment (MoCA)

The Montreal Cognitive Assessment (MoCA) is a brief screening tool used to assess cognitive functioning [32]. The MoCA assesses multiple cognitive domains including attention and concentration, executive functioning, memory, language, visuo-constructional skills, conceptual thinking, calculation and orientation. Scores ranged from 0 to 30 with scores of 26 or higher considered within normal range. Given that many psychiatric syndromes are associated with cognitive impairment (e.g. schizophrenia), we did not exclude participants scoring less than 26. Results are reported for those with scores above and below this threshold.

#### Simple physical activity questionnaire

**Fig. 1:**
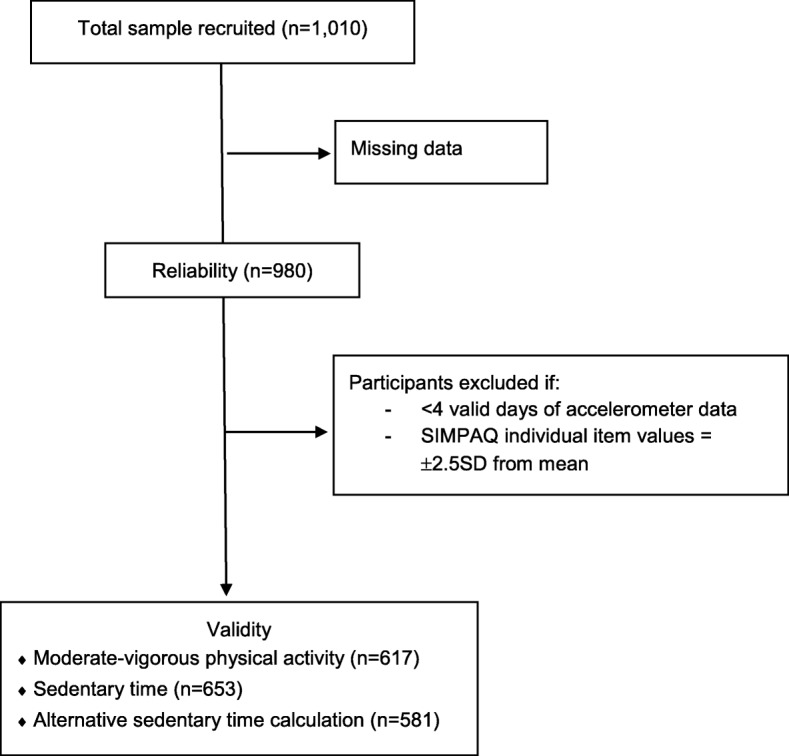
Flow diagram of participants and analyses

The 5-item SIMPAQ required people being interviewed to account for time spent in bed overnight (box 1), time sedentary, including napping (box 2), time spent walking (box 3), time spent exercising (box 4) and time engaged in incidental activity (box 5), averaged over the past seven-day period (see Fig. 1). The sum of the hours recorded in the five SIMPAQ boxes should add to approximately 24-h, providing interviewers with an opportunity to clarify with participants if significant under or over-reporting has occurred (e.g. < 18 h or > 30 h of estimated time). For an estimate of total self-reported moderate-vigorous physical activity (MVPA) time, time spent walking (box 3) and exercising (box 4) were combined to provide total MVPA (hours per week).

#### Percentage of 24-h period accounted for by SIMPAQ items

The SIMPAQ was designed to capture activity over a representative 24-h period from the previous 7-days. By summing Boxes 1 through 5, the total hours accounted for should equal approximately 24. To evaluate how well this was achieved in the current study, we calculated the fraction of time accounted for by using the following formula:

sedentary time (box 2) + walking time (box 3) + exercise time (box 4) + incidental activity time (box 5)

______________________________________________

24 – time in bed (box 1)

#### Accelerometer – Actigraph GT3/x

Participants were asked to wear a tri-axial accelerometer (Actigraph GT3x or GT3x+; ActiGraph LLC, Fort Walton Beach, FL) on the right hip during waking hours for a period of seven consecutive days to objectively assess physical activity. Accelerometers record raw acceleration data (at a sampling interval of 60 s epochs) that is converted into objective activity measures such as step counts. Participants were shown how to wear the device on the right hip using either a belt clip or elastic waist band. After the seven-day period participants returned the device and again completed the SIMPAQ for comparison with Session 1 data. Prior to Actigraph devices being issued to participants they were initialised using the online portal. Each participant was setup in CentrePoint and sex, age and weight were entered and the device allocated to the subject. Accelerometry data were retrieved from the device using CentrePoint, a secure online portal designed and distributed by Actigraph specifically for multi-site study co-ordination. ActiLife v6.13.3 software was used to extract data from CentrePoint and derive variables to be used in the calculation of validity between accelerometry data and SIMPAQ items. Participant data were included for analysis if at least eight hours of valid wear time were available for at least four days. Non wear time was defined as at least 60 min of consecutive zeroes, allowing for spike level of 100 counts per minute [33]. We followed Freedson et al. [34] to classify activity intensity using cutpoints for time spent in sedentary (< 100 cpm), light (100–2019 cpm), moderate (2020–5998 counts/ min), and vigorous intensity (> 5999 cpm) activity [34].

### Data analysis and cleaning

Non-parametric Spearman correlation coefficients were calculated as the primary measure of agreement between assessment time points (Session 1 and Session 2) (test-retest reliability), and between the SIMPAQ data and accelerometer counts (evidence of validity). Agreement between the SIMPAQ and accelerometer data was also assessed through Bland-Altman mean-difference plots with 95% limits of agreement. Intraclass correlation coefficients (ICC) along with 95% confidence intervals were also calculated. Analyses were conducted both with all valid data, and excluding outliers defined as those with SIMPAQ values that were greater or less than 2.5 SD from the mean for that item. Results are reported for the entire sample with available data and stratified by cognitive function as assessed by the MoCA and psychiatric symptom severity derived from the DSM – Cross-cutting tool. The sample were also stratified according to specific diagnoses, and those with psychiatric comorbidity. Income status, treatment setting, sex, age, body mass index (BMI) and smoking status data were analysed separately. Data were analysed using SPSS v24.

#### Reliability

Test re-test reliability was determined using Spearman Rho correlation coefficients between SIMPAQ items at Session 1 and Session 2. Given that the SIMPAQ asks responders to report activity from the previous seven-day period, and the potential for hospital admission to impact physical activity levels, only data from outpatients were utilised for reliability calculations.

#### Validity

To provide evidence for the validity of the SIMPAQ questionnaire, Spearman correlation coefficients were calculated for MVPA as assessed by the SIMPAQ (box 3 + box 4) and MVPA as recorded by the accelerometer, and for sedentary time (SIMPAQ box 2) against the accelerometer.

## Results

### Demographics

In total, data were collected from 1010 participants recruited from 23 countries. More than half of the sample of participants were male (56%), from a high income country (77%), between 25 and 54 years old (71%), current smokers (60%), overweight or obese (60%; mean BMI = 27.1 SD 5.8), did not complete any paid employment in the previous seven-day period (70%) and were recruited from an inpatient facility (53%) (See Table 1). Overall, there was significant psychiatric comorbidity (34%). Of those with a single diagnosis, the most prevalent condition was schizophrenia (23%) followed by depression (16%) and bipolar disorder (14%). In total, 65% of the sample (*n* = 648) scored greater than or equal to 26 on the MoCA indicative of normal cognitive functioning. Regarding medication usage, 56% of the sample were reported as receiving antipsychotic medication, 47% antidepressant medication and 29% were prescribed mood-stabilisers. Physical comorbidities were also recorded on the standardised assessment form with hypercholesterolemia (14%) the most commonly reported, followed by chronic pain (13%), hypertension (13%) and diabetes (6%).

**Table 1:** Demographic characteristics

		N	%
Total sample		1010	
Sex	Male	561	56
	Female	449	44
Age group	18–24 years	156	15
	25–34 years	243	24
	35–44 years	231	23
	45–54 years	238	24
	55–65 years	142	14
Diagnosis	Psychiatric Comorbidity	343	33
	Schizophrenia only	233	23
	Bipolar disorder only	145	14
	Depressive disorder only	159	16
	Other	130	14
Psychotropic Medication	Antipsychotic	562	56
	Antidepressant	477	47
	Mood-stabiliser	290	29
Cognitive ability	Normal (> = 26)	648	65
	impaired (< 26)	354	35
Treatment setting	Inpatient	537	53
	Outpatient	469	47
Smoking status	Smoker	611	60
	Non-smoker	399	40
Body mass index (BMI)(kg/m^2^)	Underweight (< 18.5)	32	4
	Desired (18.5–24.99)	305	36
	Overweight (25–29.99)	267	31
	Obese I (30–34.99)	171	20
	Obese II (35–39.99)	50	6
	Obese III (40–44.99)	27	3
Region	Europe	507	50
	Asia	249	25
	Oceania	144	14
	Americas	100	10
	Africa	10	1
	High income	777	77
Country income status	Other (lower-upper middle income)	233	23

### Percentage of 24-h period accounted for by SIMPAQ

In the overall sample, 70% of a standard 24-h period was accounted for by the SIMPAQ. This did not vary within any subgroups, with 70–80% of a 24-h time period consistently accounted for across region, country income status, diagnostic group, cognitive ability, smoking status and age.

### Reliability

Test-retest repeatability was assessed in outpatients (see Table 2). For these participants (*n* = 452), Spearman correlation coefficients were 0.75, *p* < 0.001 (box 1 – time spent in bed), 0.69, p < 0.001 (box 2 – time spent sedentary), 0.76, p < 0.001 (box 3 – time spent walking), 0.76, p < 0.001 (box 4 – time spent exercising) and 0.63, p < 0.001 (box 5 – time spent in incidental activity), indicating acceptable to good reliability.

**Table 2:** Test-retest reliability of SIMPAQ items (Spearman Rho correlation coefficients) in outpatients

	N	Box 1: Time in Bed	Box 2: Sedentary time	Box 3: Walking time	Box 4: Exercise time	Box 5: Incidental activity time
Total outpatients	452	0.75	0.69	0.76	0.76	0.63
Outpatients by country income status						
high income	323	0.8	0.68	0.59	0.69	0.58
other (lower-upper middle income)	131	0.7	0.49	0.74	0.84	0.81

All p’s < 0.001

*N’s for treatment setting and country income status do not equal total sample due to missing demographic data

### Evidence of validity

To assess validity, only participants with a minimum of four valid days of accelerometer data were included. In addition, for each individual SIMPAQ item, participants who scored ±2.5SD from the mean were excluded (Fig. 1; n’s for individual items range from *n* = 581 to *n* = 653).

### Moderate-to-vigorous physical activity

The Spearman rho correlation coefficient between the two measures for moderate-to-vigorous physical activity was 0.25 for the entire sample with available data (*n* = 617, p < 0.001; ICC = 0.23, 95% CI 0.01 to 0.34) (Table 3). For those with higher MoCA scores, the Spearman rho correlation coefficient was 0.32 (*n* = 401, p < 0.001) and for those with lower MoCA scores, 0.10 (*n* = 210, *p* = 0.17). Validity was lower in high-income countries, and this was most evident in data from European sites (Table 3). High-income countries in Oceania had larger correlations than the full sample. Larger correlations were observed in current smokers than those who were non-smokers. Evidence of validity was lower in those aged 55–65. Correlations were higher for those who were obese compared to those who were normal weight or overweight. Participants who were inpatients at the time of assessment had lower correlations than those who were outpatients. Those with psychiatric comorbidity showed comparable correlations, while a higher correlation was found in those with a diagnosis of depression in comparison with those with a diagnosis of schizophrenia. There was no difference in correlations as a consequence of psychiatric symptom severity.

**Table 3:** Correlations between MVPA assessed via the SIMPAQ and accelerometry

	N	Spearman rho	p
Total sample	617	0.25	< 0.001
Sex			
male	340	0.25	< 0.001
female	274	0.23	< 0.001
Treatment setting			
inpatient	346	0.09	0.11
outpatient	264	0.43	< 0.001
Country income status			
high income	480	0.12	0.01
other (lower-upper middle income)	134	0.26	0.002
Cognitive ability			
normal (> = 26)	401	0.32	< 0.001
impaired (< 26)	210	0.10	0.17
Diagnosis			
psychiatric comorbidity	212	0.25	< 0.001
schizophrenia only	130	0.13	0.14
bipolar disorder only	78	0.23	0.04
depressive disorder only	112	0.33	< 0.001

*All participants with available data were included in each analysis

**Fig. 2:**
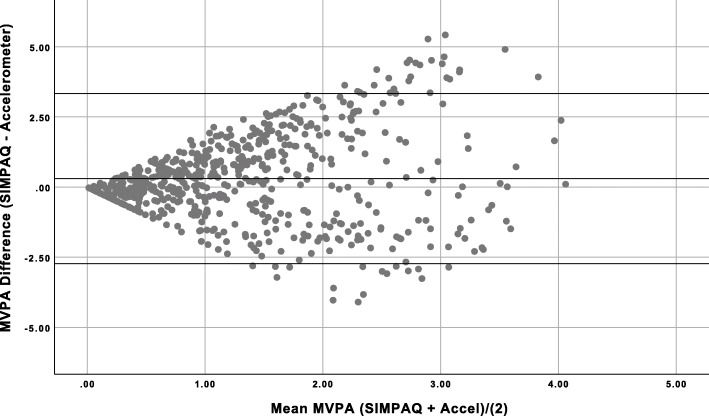
Bland-Altman plot of absolute difference between MVPA assessed via SIMPAQ and accelerometery derived MVPA estimate

The Bland-Altman plot for MVPA (Fig. 2) indicates less agreement between the two measures with higher values of MVPA.

### Sedentary time

**Fig. 3:**
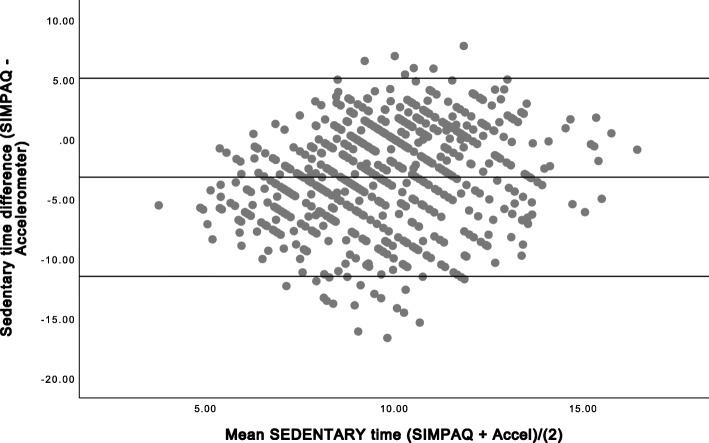
Bland-Altman plot of absolute difference between sedentary time assessed via SIMPAQ and accelerometery derived estimate

The Spearman rho correlation coefficient was not statistically significant for the entire sample with available data (rho = 0.02, *n* = 653; *p* = 0.6, ICC = 0.01, 95% CI − 0.15 to 0.15) (Table 4). For those with a higher MoCA score, the Spearman rho correlation coefficient was 0.06 (*n* = 431) and for those with lower MoCA scores, − 0.06 (*n* = 215). Psychiatric comorbidity did not impact the magnitude of the correlation and there was no difference in correlations as a consequence of psychiatric symptom severity. There was considerable variability in the observed correlation coefficients between SIMPAQ box 2 and sedentary time as assessed by the accelerometer. The correlation was lower in high income countries, and highest in Oceania and Asia. Correlations were similar for smokers and non-smokers, and higher in those who were older, overweight or obese and outpatients. The Bland-Altman plot for sedentary time (Fig. 3) showed no evidence of bias with higher or lower values of sedentary time as assessed by the two measures.

**Table 4:** Correlations between sedentary behaviour assessed via the SIMPAQ and accelerometry

	N	Spearman rho	p
Total sample	653	0.02	0.57
Sex			
Male	360	0.08	0.12
Female	274	−0.08	0.19
Treatment setting			
inpatient	377	−0.08	0.14
outpatient	269	0.14	0.02
Country income status			
high income	518	0.04	0.38
other (lower-upper middle income)	132	0.11	0.23
Cognitive ability			
normal (> = 26)	431	0.06	0.22
impaired (< 26)	215	−0.06	0.41
Diagnosis			
psychiatric comorbidity	220	0.03	0.71
schizophrenia only	140	0.04	0.66
bipolar disorder only	84	0.08	0.47
depressive disorder only	123	−0.03	0.72

*All participants with available data were included in each analysis

### Alternative method for calculating sedentary time

Given that self-report questionnaires are likely to lead to underestimates of sedentary behaviour, and given that the average percentage of time accounted for by the SIMPAQ as a percentage of 24-h (70–80%), we derived an alternative method of scoring sedentary time from the SIMPAQ. We summed the scores of time spent in bed (box 1), time spent walking (box 3), time exercising (box 4) and time incidental activity (box 5), which we defined as non-sedentary time. We subtracted this figure from 24-h to provide an alternative estimate of sedentary behaviour. Evidence of validity for this alternative method was statistically significant for the overall sample (rho = 0.19, *n* = 581, *p* < 0.001; ICC = 0.29, 95% CI 0.17 to 4.0 (Table 5).

**Table 5:** Correlations between sedentary behaviour assessed via the SIMPAQ and accelerometry, using the alternative SIMPAQ scoring method

	N	Spearman rho	p
Total sample	581	0.19	< 0.001
Sex			
Male	319	0.20	< 0.001
Female	259	0.18	< 0.01
Treatment setting			
inpatient	331	0.22	< 0.001
outpatient	243	0.18	< 0.01
Country income status			
high income	474	0.24	< 0.001
other (lower-upper middle income)	104	−0.14	0.16
Cognitive ability			
normal (> = 26)	384	0.15	< 0.01
impaired (< 26)	191	0.25	< 0.001
Diagnosis			
psychiatric comorbidity	199	0.27	< 0.001
schizophrenia only	126	0.26	< 0.01
bipolar disorder only	74	0.04	0.76
depressive disorder only	112	0.09	0.32

*All participants with available data were included in each analysis

## Discussion

This study examined the test-retest reliability and evidence of validity of a novel, brief, interview-based, self-reported physical activity measure, designed for routine clinical use within psychiatric settings. In a large diverse sample of psychiatric patients, ascertained across a variety of treatment settings and including a range of psychiatric diagnoses, with substantial representation from low- and middle- income countries, we found that the SIMPAQ was a reliable tool for assessing physical activity and sedentary behaviour. Evidence of validity for MVPA was higher for outpatients than inpatients and was comparable to that reported in general population samples [35, 36] and in smaller cohorts of people with mental illness [23].

In physical activity research, correlation coefficients between self-report and objective measures of physical activity of 0.3 are often reported as acceptable evidence of validity [35–39]. This limited shared variance reflects the challenges associated with both self-report questionnaires and accelerometers when assessing physical activity in the general population. Given that the correlations found for the SIMPAQ were not substantially lower than those deemed acceptable in general population samples, attests to the utility of the SIMPAQ in people with mental illness who can experience a range of additional challenges e.g. psychiatric symptoms and cognitive impairment.

Correlations were lower for those with MoCA scores below the usual cut-off indicative of cognitive impairment. We explicitly did not use the MoCA score as an exclusion criterion considering that a number of psychiatric syndromes are characterised by cognitive impairment, e.g. schizophrenia. While the reliability of the SIMPAQ was largely unaffected by cognitive capacity, it is evident that those with lower MoCA scores had a lower correlation with objectively measured MVPA. Therefore, self-reported MVPA in those with higher levels of cognitive impairment may be less accurately reported.

For the overall sample, self-reported and objectively assessed sedentary time were not significantly correlated. Significant correlations were found for outpatients, which may reflect the statistically significant lower symptom severity (*p* < 0.001) and greater cognitive (p < 0.001) capacity of our outpatient sample. People living with more severe mental illness may engage in high levels of sedentary behaviour, and combined with some degree of cognitive impairment, are likely to experience particular difficulty in accurately estimating sedentary time [40]. Additionally, the poor correlations between the SIMPAQ and objective measure of sedentary behaviour can be in part explained by the fact that the Actigraph was waist-mounted and therefore is not a true assessment of postural allocation (i.e. sitting or standing). Therefore low intensity activities performed while sitting or standing may have been misclassified [41].

Given the known limitations of self-reported estimates of sedentary behaviour in both the general population [41] and in people living with mental illness [25, 26], we generated an alternative method for calculating sedentary time using the SIMPAQ data (see Section 3.5). This involved summing the scores of non-sedentary time estimates (boxes 1, 3, 4 and 5) and subtracting this from 24 h. This method therefore takes into account the tendency for underreporting of sedentary behaviour and based on the correlation analysis, appears to be a more valid estimate of sedentary behaviour in the target population. Based on these results, we recommend users of SIMPAQ adopt this alternative scoring method to obtain more valid estimate of sedentary behaviour, especially among inpatients and those with high levels of cognitive impairment. Future research should also aim to investigate the validity of the SIMPAQ sedentary behaviour item using inclinometers.

The evidence of validity of the SIMPAQ as a tool to assess MVPA was comparable to other self-report measures in the general population (e.g. [36]), and results were relatively consistent across diagnoses, sex and age. Unsurprisingly, we found different levels of correlations in different settings and among different sub-groups within the sample. It should be noted that SIMPAQ was designed to be used as a clinical tool administered by health professionals regardless of training or expertise in exercise prescription or assessment. In some of the participating centres, SIMPAQ was administered by exercise specialists (e.g. physical therapists or exercise physiologists), whereas in other sites SIMPAQ was administered by staff with primary mental health qualifications (e.g. psychiatrists or psychiatric nurses). There was no evidence of greater validity in settings where exercise professionals administrated the SIMPAQ versus mental health professionals. Given the diverse backgrounds of people likely to administer the SIMPAQ, the table in Item 4 of the tool deliberately allows for either a brief summary, or a more comprehensive assessment of exercise time (e.g. by completing the entire Table) if clinically indicated or the assessor has available time.

Limitations of this study include the opportunistic sampling method that does not reflect the global diagnostic prevalence of different psychiatric disorders. While effort was made to recruit a diverse sample of participants from a range of settings including high and low income countries, there was an overrepresentation from high income, English speaking countries. Regarding the development of the SIMPAQ, in order to maximise clinical utility, we aimed to ensure that administration time was minimised and therefore comprehensive assessment of detailed aspects of physical activity such as the domain are not specifically evaluated. Another limitation is the use of accelerometers as the objective measure of physical activity. While accelerometers are cheaper and more accessible than other forms of objective measurement, they are not without limitations including the inability to assess movement associated with non-ambulatory activity (e.g. cycling and resistance training) [42].

## Conclusion

In conclusion, we demonstrated that the SIMPAQ is a reliable and valid tool to assess physical activity in people living with mental illness. SIMPAQ does not require detailed training, identifies even small amounts of activity which is useful in providing positive feedback to patients participating in physical activity interventions, is quick to administer and did not prove difficult for people with mental health problems to complete. These initial results are promising and suggest that the instrument is an appropriate tool for routine use in clinical mental health services. Assessing and promoting physical activity as a component of care within mental health services is a key means by which the physical and mental health of this population can be improved.

## Data Availability

The dataset used during the current study is available from the corresponding author on reasonable request.

## Abbreviations

BMI: Body mass index
MoCA: The Montreal Cognitive Assessment (MoCA)
MVPA: Moderate-vigorous physical activity
SIMPAQ: Simple Physical Activity Questionnaire
